# The Mechanistic Perspective of Bilobetin Protective Effects against Cisplatin-Induced Testicular Toxicity: Role of Nrf-2/Keap-1 Signaling, Inflammation, and Apoptosis

**DOI:** 10.3390/biomedicines10051134

**Published:** 2022-05-13

**Authors:** Walaa A. Negm, Aya H. El-Kadem, Ismail A. Hussein, Moneerah J. Alqahtani

**Affiliations:** 1 Department of Pharmacognosy, Faculty of Pharmacy, Tanta University, Tanta 31527, Egypt; 2 Department of Pharmacology and Toxicology, Faculty of Pharmacy, Tanta University, Tanta 31527, Egypt; 3 Department of Pharmacognosy and Medicinal Plants, Faculty of Pharmacy (Boys), Al-Azhar University, Cairo 11884, Egypt; ismaila.hussein@azhar.edu.eg; 4 Department of Pharmacognosy, College of Pharmacy, King Saud University, P.O. Box 2457, Riyadh 11451, Saudi Arabia; mjalqahtani@ksu.edu.sa; 5 Department of BioMolecular Sciences, Division of Pharmacognosy, School of Pharmacy, University of Mississippi, Oxford, MI 38677, USA

**Keywords:** bilobetin, *Cycas thouarsii*, caspase-3, cisplatin, keap-1, Ki67

## Abstract

Cisplatin (CP) is a productive anti-tumor used to treat numerous tumors. However, multiple toxicities discourage prolonged use, especially toxicity on the reproductive system. This experiment was mapped out to determine the potential therapeutic impact of Bilobetin on CP-induced testicular damage. Herein, Bilobetin was isolated from *Cycas thouarsii* leaves R. Br ethyl acetate fractions for the first time. A single dose of CP (7 mg/kg, IP) was used to evoke testicular toxicity on the third day. Rats were classified into five groups; Normal control, Bilobetin 12 mg/kg, Untreated CP, and CP treated with Bilobetin (6 and 12 mg/kg, respectively) orally daily for ten days. Bilobetin treatment ameliorated testicular injury. In addition, it boosted serum testosterone levels considerably and restored relative testicular weight. Nevertheless, apoptosis biomarkers such as P53, Cytochrome-C, and caspase-3 decreased significantly. Additionally, it enhanced the testes’ antioxidant status via the activation of Nrf-2, inhibition of Keap-1, and significant elevation of SOD activity in addition to a reduction in lipid peroxidation. Histopathologically, Bilobetin preserved testicular architecture and improved testicular immunostaining of Ki67 substantially, showing evidence of testicular regeneration. Bilobetin’s beneficial effects on CP-induced testicular damage are associated with enhanced antioxidant effects, lowered apoptotic signals, and the restoration of testes’ regenerative capability. In addition, Bilobetin may be used in combination with CP in treatment protocols to mitigate CP-induced testicular injury.

## 1. Introduction

Cisplatin (CP) has been used to treat a variety of cancers for over 50 years, including breast, ovarian, testicular, and bladder tumors [1]. Cisplatin is a potent anti-tumor agent, although it harms the kidneys, liver, gut, and testis [2]. CP works because it binds to purine bases in DNA, causing DNA strand breaks and cell death [3]. Apoptosis, inflammation, and oxidative stress have also been implicated as significant causes of CP’s harmful effects on tissues [4]. Cisplatin-induced testicular damage is a significant barrier to its application as an anticancer agent [5].

Cisplatin induces severe testicular damage by impairing Leydig cell activity, decreasing testosterone production, and inducing germ cell apoptosis, according to several studies. In addition to DNA damage, the enhanced production of reactive oxygen species (ROS) is the major mechanism of CP-induced testicular injury [6].

As a result, temporary or permanent infertility is one of the most common issues following CP applications [4]. As a result, protecting the testes against the harmful effects of CP has become critical.

Several natural compounds, such as flavonoids, biflavonoids, volatile oils, and phenolic acids, have been shown to reduce oxidative stress and have anti-inflammatory characteristics, which may help prevent CP-induced testicular damage [3,7,8,9]. In addition, natural product research is widely considered a powerful method for discovering effective, safe, and convenient medications [9].

Bilobetin, a natural biflavonoid molecule derived from some gymnosperm plants [10,11,12,13], has a diverse set of pharmacological effects involving antioxidation, anticancer, antibacterial, antifungal, anti-inflammatory, antiviral, and osteoblast differentiation promotion [14,15]. Unlike terpenes, bioflavonoids such as Bilobetin have low oral bioavailability due to the first pass effect and glucuronidation [16]. However, few studies have been conducted on Bilobetin and other related biflavonoids [12,17,18].

To the best of our knowledge, this study is the first to isolate Bilobetin from *Cycas thouarsii* R.Br, explore its potential mitigative effects against CP induced testicular toxicity, and elucidate the possible underlying mechanisms of such beneficial effects in vivo.

## 2. Materials and Methods

### 2.1. Plant Extraction and Bilobetin Isolation

The *Cycas thouarsii* R.Br. Leaves were obtained from El-Abd Nursery in Giza in Jan 2017. Dr. Esraa Ammar, Plant Ecology Department, Tanta University, kindly confirmed plant identifications. A voucher specimen (PGG-W-004) was kept at the Pharmacognosy Department. The powdered leaves (1750 g) were extracted with methyl alcohol (four times × 5 L). The extract was concentrated using a rotary evaporator to acquire the total extract residue. The total methanolic extract (70 g) was resuspended in MeOH: H_2_O (50%), and then partitioned with *n*-hexane, dichloromethane (DCM), ethyl acetate, and then *n*-butanol saturated with H_2_O, yielding different fraction residues, respectively [11].

Ethyl acetate fraction (3.8 g) was column chromatographed CC (ϕ 2.5 × 70 cm, 100 g silica, collected fraction 30 mL) using gradient elution, starting with DCM, and then CH_3_OH was used to obtain five fractions (E1: E5). Fr. E1 (DCM–CH_3_OH; 96:4 eluate), Fr. E2 (DCM– CH_3_OH; 94:6 eluate), Fr. E3 (DCM–CH_3_OH; 92:8 eluate), Fr. E4 (DCM– CH_3_OH; 90:10 eluate), and Fr. E5 (DCM–CH_3_OH; 86:14 eluate). Fr. E1 (1.32 g) was chromatographed using silica gel, sub-fractions (eluted with CHCl_3_–CH_3_OH; 91:9) were collected and then purified using Sephadex LH-20 eluted with 100% CH_3_OH to obtain a pale-yellow amorphous powder of Compound (I).

### 2.2. Animals

Forty adult male albino rats obtained from the animal house of Cairo University, Egypt, weighing 170–210 gm, were utilized. They were fed standard pellet chow (EL-Nasr Chemical, Egypt) and allowed free access to water. Rats were housed for one week before the experiment for acclimatization.

All protocols and procedures were carried out in accordance with the guidelines for the care and use of laboratory animals approved by the Research Ethics Committee (Tanta University, NO: TP/RE/9/21-Pr-005).

### 2.3. Chemicals and Spectral Techniques

Mylan Pharmaceuticals Co. obtained cisplatin (50 mg/50 mL) injection. All other chemicals and solvents were purchased from Sigma-Aldrich unless otherwise mentioned. For CC, we employed Silica gel F254 (Merck, 70–230 mesh) and Sephadex LH-20 (Sigma–Aldrich Chemical Co., St. Louis, MO, USA).

A JEOL ECA500-II-NMR spectrometer recorded NMR spectra at 500 MHz for ^1^H and 125 MHz for ^13^C. DMSO-*d*_6_ was utilized to dissolve the NMR sample. The chemical shifts were normalized using solvent resonances. Thermo Scientific’s ISQ Quantum Access MAX Triple Quadrupole system, Xcalibur 2.1 software, and USA Mass Spectrometer were utilized for the ESI-MS.

### 2.4. Experimental Design

Cisplatin-induced testicular damage was induced by CP as described previously [7,19]. Forty male Rats were grouped randomly into five groups (8/rats each).

Group I: Daily, the control group received normal IP saline.

Group II: The Bilobetin group was administered Bilobetin (12 mg/kg) (dissolved in phosphate-buffered saline and injected IP Daily for ten days.

Group III: The CP group rats were treated with a single IP injection of 7 mg/kg of CP, which was previously used to induce testicular damage [3,7,19,20,21].

Group IV and V: CP+ Bilobetin groups were administered Bilobetin (6, 12 mg/kg respectively) [13] and dissolved in phosphate-buffered saline-injected IP daily for ten days and received a single dose of IP of CP 7 mg/kg on the third day.

### 2.5. Sample Collection

On the 11th day, all animals were weighed and slaughtered under light ether anesthesia. For hormonal testing, blood samples were taken by utilizing heart puncture. Testes were dissected and weighed right after blood was drawn. Following that, a portion of the testis was fixed in 4% paraformaldehyde solution for histology, while the left testis was maintained in liquid nitrogen at −70 °C for further evaluation.

### 2.6. Determination of Testis Body Weight Ratio

The testis body weight ratio is estimated by dividing the weight of the testes in gm by the final weight of the body and multiplying it by 100 [22].

### 2.7. Assessment of Serum Testosterone and Cytochrome-c

To evaluate Bilobetin 6 and 12’s influences on cisplatin testicular toxicity, Rat Testosterone ELISA and Rat Cytochrome-C ELISA kits (CUSABIO TECHNOLOOGY LLC) were used to estimate Testosterone and Cytochrome-C level following the manufacturer’s protocol. In brief, all reagents and samples were prepared as directed by the kit’s instructions. The blank well was set, 50 µL of standard or sample was added per well, and then 50 µL of antibody was added, mixed, and incubated as directed. The plate was washed in triplicate by a wash buffer, and then 50 µL of substrate A and substrate B was added to each well and incubated for 15 min. Then, the stop solution was added, mixed gently, and finally, optical density was determined at 450 nm.

### 2.8. Measurement of Lipid Peroxidation

The measurement of lipd peroxidation was performed by measuring malondialdehyde (MDA) levels in the testis tissue homogenate using (Biodiagnostic, Giza, Egypt) kits. In brief, 0.2 mL of the tissue homogenate or standard (10 nmol/mL) was mixed with 1 mL of chromogen (25 mmol/L) in a test tube and heated in a boiling water bath for 30 min, and then it was cooled, mixed, and absorbance was measured at 534 nm.

### 2.9. Measurement of SOD Activity

The superoxide dismutase enzyme activity in the testis homogenate was measured following the manufacturer’s instructions and utilizing a commercially available kit obtained from Biodiagnostic, Giza, Egypt. In brief, 0.1 mL of tissue homogenate was mixed with 1 mL of working reagent (Phosphate buffer pH 8.5 (50 Mm/L), Nitro blue tetrazolium, and NADH (1 Mm/L) in ratio 10:1:1). The reaction was initiated by adding 0.1 mL phenazine methosulphate (PMS) (0.1 Mm/L). The increase in absorbance was measured at 560 nm for 5 min for the control and sample.

### 2.10. qRT-PCR forVCAM, NrF-2, Keap-1, IL-0, α-SMA, and P53 Genes

For total RNA purification from testicular samples, the TRIzol reagent (Life Technologies, Inc, Carlsbad, CA, USA) was utilized. In a two-step technique RT-PCR process, 1 μg of total RNA was reverse-transcribed into single-stranded complementary DNA using the QuantiTects Reverse Transcription Kit (Qiagen, Germantown, MD, USA) and a random primer hexamer. Maximas SYBR Green/Fluorescein qPCR Master Mix was used to amplify C-DNA amplicons using particular primers produced according to the manufacturer’s procedure (Table S1).

Each sample was tested in duplicate with real-time PCR, and the mean values of the duplicates were used for further analysis. Finally, the 2^−ΔΔCT^ method was measured relative to mRNA expression, and then it was normalized at GAPDH [23,24].

### 2.11. Histopathological Examination of Testis Sections

Paraffin blocks of the liver were sectioned at 4 μm thick and stained with hematoxylin and eosin (H&E) and examined under a light microscope.

### 2.12. Immunohistochemical Staining of Ki67 and Caspase-3

The immunohistochemical staining steps for ki67 and caspase-3 were conducted using their active antibodies ki67 and caspase-3 (ABclonal Technology, Woburn, MA, USA). The staining procedure was at a magnification of 100× in all fields of tissue slices. According to the percentage of Ki67 positive cells (nuclear staining), caspase-3 positive cells (nuclear with or without cytoplasmic staining), and immunohistochemical staining results were scored according to the method described by Sherif et al. [25]. Regarding Ki67 staining, the basal cell layer staining of seminiferous tubules was excluded (normal proliferation). Immuno-stained slides were image analyzed using Image J software. The staining scores were calculated by the percentage of positive cells within 1000 cells being counted on each slide in the area of maximum staining per 10 high power fields after background subtraction.

### 2.13. Statistical Analysis

The data are provided as a mean ± standard deviation. Regression analysis was performed on all calibration curves, producing correlation coefficients. A one-way analysis of variance (ANOVA) was utilized to compare different groups, followed by a Tukey–Kramer post hoc test. *p* < 0.05 was used as the significant level. The statistical analysis was carried out using Prism version 9 (GraphPad Software, Inc., San Diego, CA, USA).

## 3. Results

### 3.1. Phytochemical Investigation

#### Structure Elucidation of Bilobetin

**Figure 1:**
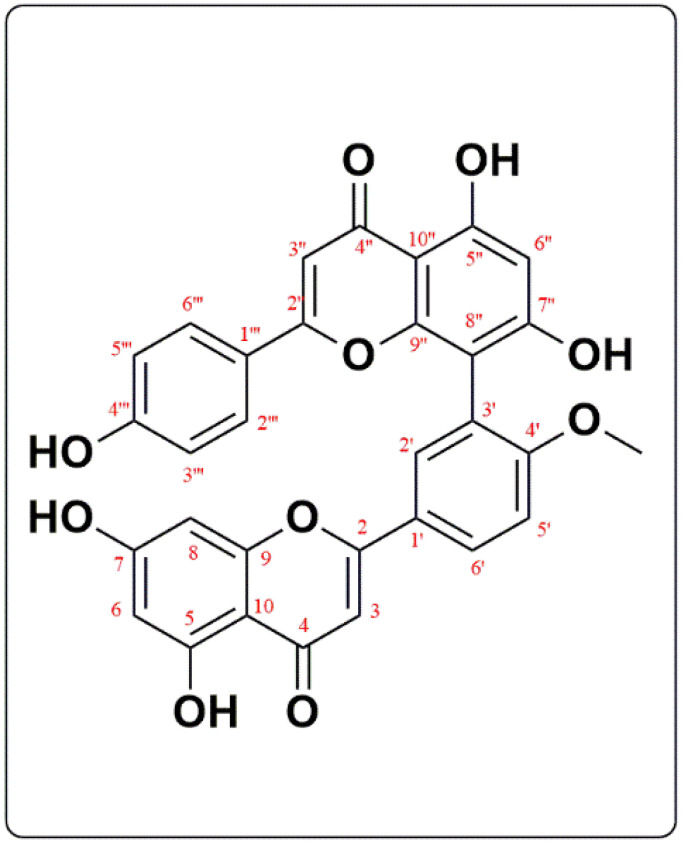
Chemical structure of Compound I (Bilobetin or 4′-*O*-methyl amentoflavone).

Compound (I) was identified as 4’-*O*-methyl amentoflavone or Bilobetin. Bilobetin is obtained as a light-yellow amorphous powder. Its UV, ESI-MS, ^1^H, and ^13^C-NMR data were compared to those described in the literature [12]. Bilobetin has a UV λ_max_ (MeOH) of 241, 298, and 380 and ESI-MS *m*/*z* 551.09 for [M-H]^-^ with a molecular formula of C_31_H_20_O_10_. Figure 1 depicts the chemical structure of Bilobetin, while the results of ^1^H-NMR (DMSO-*d*_6_, 500 MHz) and ^13^C-NMR (DMSO-*d*_6_, 125 MHz) are listed in Table 1.

### 3.2. Biological Investigation

#### 3.2.1. Effects of Bilobetin on Testicular Weight Changes

The findings revealed that the Bilobetin-only treated group has comparable results to the normal control group regarding all assessed parameters, confirming that Bilobetin treatment does not have any harmful effects on testicular functions. Relative to the control, the testicular weight of the rats in the CP-treated group was considerably lower (35.8%). Bilobetin co-treatment, on the other hand, substantially reduced testicular weight loss in CP-treated rats (17.8, 41.64%, respectively) (*p*< 0.05). In addition, rats in the Bilobetin 12 group had similar testicular weights to those in the control group (Table 2).

Cisplatin induced a marked decrease in testes body weight ratio (25.69%) compared to the control group, which is significantly increased by Bilobetin 6 and 12 co-treatments (18.3 and 28.9%), respectively. The effect was more pronounced in Bilobetin 12 (*p* < 0.05) (Table 2).

#### 3.2.2. Effects on Serum Testosterone Level

**Figure 2:**
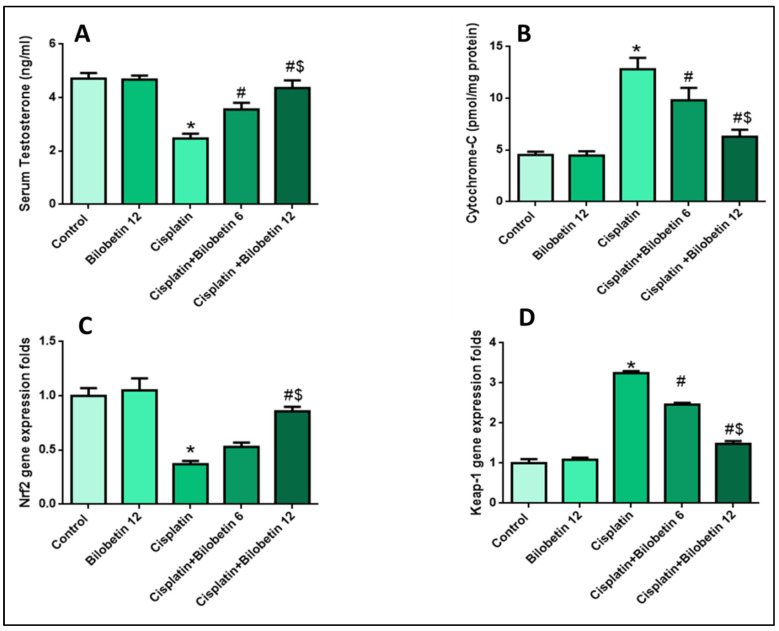
Effects of Bilobetin treatment on (**A**) Serum Testosterone level, (**B**) Cytochrome-C level, (**C**) Nrf2 gene expression level, and (**D**) Keap-1 gene expression level in CP-induced testicular toxicity in rats. CP-induced testicular damage was induced by a single i.p. injection of 7 mg/kg of CP on day 3. Rats were grouped randomly into the control group. Bilobetin group was administered Bilobetin (12 mg/kg) i.p. daily for ten days; untreated CP group and CP groups were treated with Bilobetin (6,12 mg/kg, respectively) i.p. daily for ten days and a single dose of i.p. injection of CP 7 mg/kg at day 3. Data expressed as mean ± SD (n = 8/group). Significant difference vs. * respective control; ^#^ respective CP group; ^$^ respective CP+ Bilobetin 6 group each at *p* < 0.05.

As indicated in Figure 2A, CP treatment caused a considerable reduction in serum testosterone levels (47.55%) relative to the control. In comparison, Bilobetin co-treatment improved the lowered testosterone levels caused by CP substantially (44.12 and 76.51%, respectively, in Bilobetin 6 and 12 groups) (*p* < 0.05) (Figure 2A).

#### 3.2.3. Effects on Cytochrome-C Release in the Cytosol

The Cytochrome-C concentration in cytosol increased substantially in CP-treated rats (183.62%) in comparison to the control group. Bilobetin 6 and 12 co-treatment significantly decreased Cytochrome-C liberation from the mitochondria. They decreased their concentration in the cytosol (23.4 and 50.93%, respectively) relative to the CP group. The Bilobetin 12 group had a more noticeable effect (*p* < 0.05) (Figure 2B).

#### 3.2.4. Effects on Testicular Oxidative Stress Markers

Table 3 reflects significant oxidative stress in the CP group. CP induced a marked elevation of testicular lipid peroxidation manifested by a major increase in MDA content (80.25%) compared to the normal control. Moreover, CP showed pronounced suppression of SOD activity (50.81%) in the testicular tissue compared to the normal group. Bilobetin co-treatment mitigated oxidative stress and improved testicular antioxidant capacities. It significantly decreased MDA levels (27.38 and 41.74%, respectively) compared to the CP group. Interestingly, Bilobetin 12 can nearly diminish MDA elevation. Results showed that Bilobetin caused about a 24.92 and 87.39% increase in SOD activity (*p* < 0.05) (Table 3).

#### 3.2.5. Effects on Testicular Nrf2 Gene Expression

In the current study, CP significantly downregulated testicular Nrf2 (63%) relative to the control group. Bilobetin co-treatment upregulated Nrf2 mRNA expression (43.24, 131.62%) relative to CP group (Figure 2C, *p* < 0.05).

#### 3.2.6. Effects on Testicular Keap-1 Gene Expression

Figure 2D showed that the CP group experienced a significant increase in keap-1 (225%) levels compared to the control. Bilobetin 6 and 12 co-treated groups considerably decreased keap-1 expression levels (24.30 and 54.46%, respectively) relative to the CP group, with a more substantial effect in the Bilobetin 12 group (Figure 2D, *p* < 0.05).

#### 3.2.7. Effects on Testicular VCAM Gene Expression

**Figure 3:**
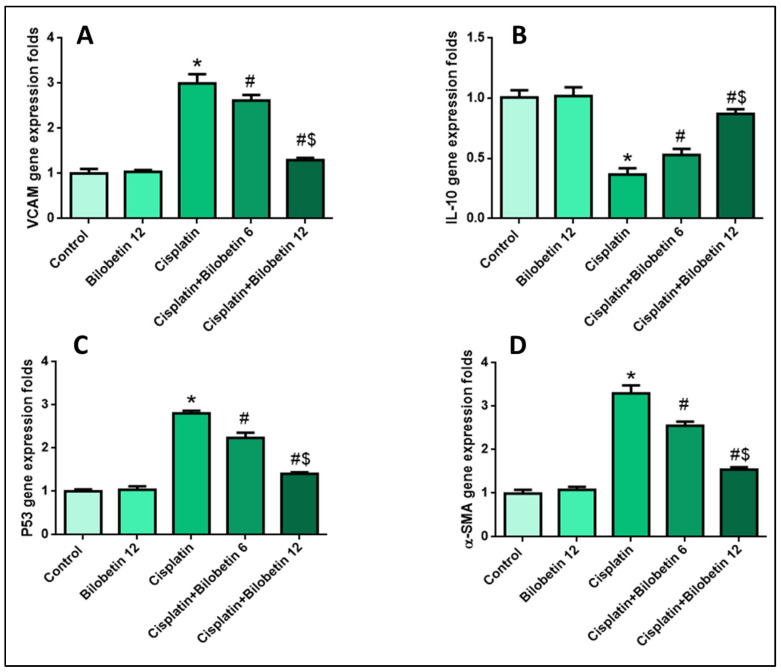
Effects of Bilobetin treatment on (**A**) VCAM gene expression level, (**B**)IL-10 gene expression level, and (**C**) P53 gene expression level. (**D**) α-SMA gene expression level. CP-induced testicular damage was induced by a single i.p. injection of CP 7 mg/kg on day 3. Rats were grouped randomly into the control group. Bilobetin group was administered Bilobetin (12 mg/kg) i.p. daily for ten days; untreated CP group and CP groups were treated with Bilobetin (6, and 12 mg/kg respectively) i.p. daily for ten days and a single dose of i.p. injection of CP 7 mg/kg at day 3. Data expressed as mean ± SD (*n* = 8/group). Significant difference vs. * respective control; ^#^ respective CP group; ^$^ respective CP+ Bilobetin 6 group each at *p* ˂ 0.05.

Figure 3A showed that the CP group caused a prominent increase in VCAM (200%) level expressions compared to the control. Bilobetin 6 and 12 co-treated groups significantly decreased VCAM expression levels (12.76 and 56.66 %, respectively) relative to the CP group, with a more significant effect in the Bilobetin 12 group (Figure 3A, *p* < 0.05).

#### 3.2.8. Effects on Testicular IL-10 Gene Expression

Cisplatin significantly suppressed testicular IL-10 expression levels (63.4%) relative to the control group. Bilobetin co-treatment markedly upregulated IL-10 mRNA expression (44.8 and 137.7%) compared to the CP group (Figure 3B, *p* < 0.05).

#### 3.2.9. Effects on Testicular P53 Gene Expression

Figure 3C showed that the CP group significantly upregulated P53 expression (181%) levels compared to the control. Bilobetin 6 and 12 co-treated groups considerably decreased P53 expression levels (20.28 and 49.82%, respectively) relative to the CP group, with a more significant impact in the Bilobetin 12 group (Figure 3C, *p* < 0.05).

#### 3.2.10. Effects on Testicular α-SMA Gene Expression

Figure 3D showed that the CP group significantly raised α-SMA (230%) expression levels compared to the control. Bilobetin 6 and 12 co-treated groups considerably reduced α-SMA expression levels (22.72 and 53.33%, respectively) relative to the CP group, with a more significant impact in the Bilobetin 12 group (Figure 3D, *p* < 0.05).

#### 3.2.11. Effects on Immunohistochemical Staining of Ki67

**Figure 4:**
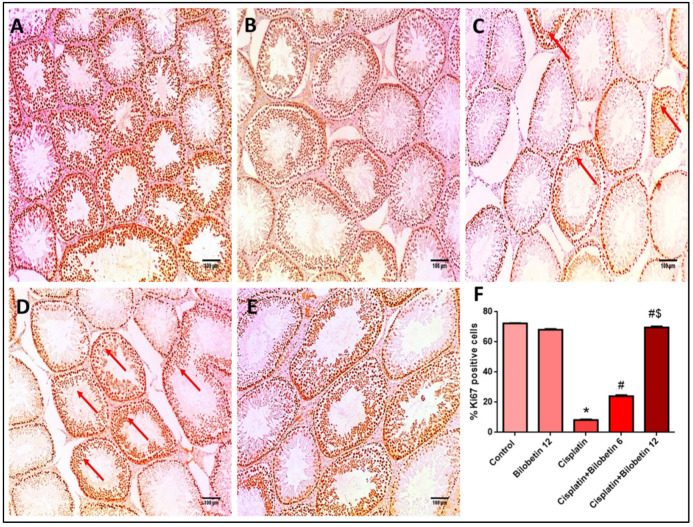
Effects of Bilobetin treatment on immunohistochemical staining of Ki67. (**A**) Section of testis of the normal control rat showed strong Ki67 staining (more than 50%) of spermatogenic cells (×100). (**B**) Section of testis of Bilobetin 12 treated group showed strong Ki67 staining (more than 50%) of spermatogenic cells (×100). (**C**) Section of testis of CP-treated rat [positive control] showed mild ki67 staining [less than 10%] of spermatogenic cells (red arrows) (×100). (**D**): Section of testis of CP+ Bilobetin 6 treated group showed moderate ki67 staining (red arrows) (10–50%) of spermatogenic cells (×100). (**E**) Section of testis of CP+ Bilobetin 12 treated group showed strong Ki67 staining (more than 50%) of spermatogenic cells (×100). (**F**) Percent of Ki67 positive cells/1000 cells per 10 high power fields. Data expressed as mean ± SD (*n* = 8/group). Significant difference vs. * respective control; ^#^ respective CP group; ^$^ respective CP+ Bilobetin 6 group each at *p* ˂ 0.05.

Figure 4A showed a section of testis of the normal control rat that showed strong Ki67 staining (more than 50%) of spermatogenic cells. While the section of testis of the Bilobetin 12 treated group showed strong Ki67 staining (more than 50%) of spermatogenic cells (Figure 4B). Moreover, the section of testis of the CP-treated rat (positive control) showed mild ki67 staining (less than 10%) of spermatogenic cells (Figure 4C). In addition, the section of testis of the Cisplatin+ Bilobetin 6 treated group showed moderate ki67 staining (10–50%) of spermatogenic cells (Figure 4D). Moreover, the section of testis of the Cisplatin+ Bilobetin 12 treated group showed strong Ki67 staining (more than 50%) of spermatogenic cells (Figure 4E). The results of immune-staining quantification revealed that both control and Bilobetin 12 groups showed strong Ki67 immunostaining. Cisplatin significantly suppressed Ki67 immunostaining by 88.59% compared to the control group. Treatment with Bilobetin 6 and 12 induced a marked increase in Ki67 staining by 191.58 and 743.6%, respectively, compared to the cisplatin group alone (Figure 4F, *p* < 0.05).

#### 3.2.12. Effects on Immunohistochemical Staining of Caspase-3

**Figure 5:**
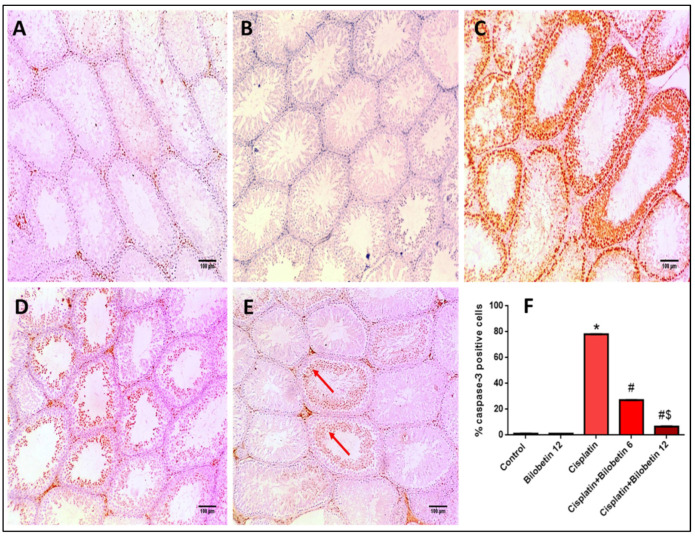
Effects of Bilobetin treatment on immunohistochemical staining of caspase-3. (**A**) Section of testis of normal control rat showed negative caspase-3 of spermatogenic cells (×100). (**B**) Section of testis of Bilobetin 12-only treated group showed negative caspase-3 staining of spermatogenic cells (×100). (**C**) Section of testis of CP treated rat (positive control) showed strong caspase-3 staining (more than 50%) of spermatogenic cells (×100). (**D**) Section of testis of CP+ Bilobetin 6 treated group showed moderate caspase-3 staining (red arrows) (less than 10%) of spermatogenic cells (×100). (**E**) Section of testis of CP+ Bilobetin 12 treated group showed mild caspase-3 staining (10–50%) of spermatogenic cells (red arrows) (×100). (**F**) Percent of caspase-3 positive cells/1000 cells per 10 high power fields. Data expressed as mean ± SD (*n* = 8/group). Significant difference vs. * respective control; ^#^ respective CP group; ^$^ respective CP+ Bilobetin 6 group each at *p* ˂ 0.05.

Figure 5A shows a section of the testis of the normal control rat that showed negative caspase-3 of spermatogenic cells. While a section of testis of the Bilobetin 12 solely treated group presented negative caspase-3 staining of spermatogenic cells (Figure 5B). A section of testis of the Cisplatin-treated rat (positive control) showed strong caspase-3 staining (more than 50%) of spermatogenic cells (Figure 5C).

In addition, a section of testis of the Cisplatin+ Bilobetin 6-treated group showed moderate caspase-3 staining (less than 10%) of spermatogenic cells (Figure 5D). Moreover, the section of testis of the Cisplatin+ Bilobetin 12-treated group showed mild caspase-3 staining [10–50%] of spermatogenic cells (Figure 5E). Results of immune-staining quantification revealed that both the control and Bilobetin 12 groups showed very weak immunostaining. Cisplatin significantly elevated caspase-3 immunostaining by 71.08-fold compared to the control group and treatment with Bilobetin 6 and 12 induced a marked suppression of caspase-3 staining by 65.21 and 91.46%, respectively, compared to the Cisplatin group alone (Figure 5F, *p* < 0.05).

#### 3.2.13. Effects on Histopathological Examination of Testicular Tissue

**Figure 6:**
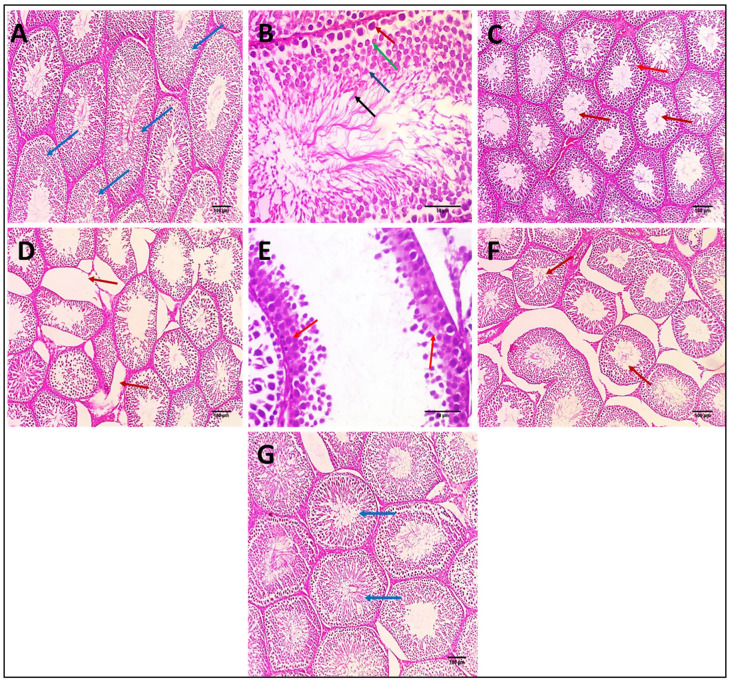
Effects of Bilobetin treatment on histopathological examination of testicular tissue. (**A**) Section of testis of normal control rat showed seminiferous tubules (blue arrows) lined by layers of spermatogenic cells and filled with spermatozoa. (H&E ×100). (**B**) Higher magnification of normal testis showed seminiferous tubule showing complete layers of spermatogenesis consisting of layers of spermatogonia (red arrow), spermatocytes (green arrow), spermatids (blue arrow), and spermatozoa (black arrows) (H&E × 400) and showed the Johnson score: Ref. [10] complete spermatogenesis with mature sperms. (**C**) Section of testis of Bilobetin 12 treated group showed seminiferous tubules (red arrows) lined by layers of spermatogenic cells and filled with spermatozoa (H&E ×100) and showed a Johnson score: Ref. [10] complete spermatogenesis with mature sperms. (**D**) Section of testis of CP treated rat (positive control) showed the destruction and disorganization of some seminiferous tubules (red arrows) (H&E × 100). (**E**) Higher magnification of CP-treated testis (positive control) showed seminiferous tubule showing many spermatocytes and no spermatids or spermatozoa (red arrows) (H&E × 400). It showed a Johnson score: Ref. [5] many spermatocytes and no spermatids or spermatozoa. (**F**) Section of testis of CP+ Bilobetin 6-treated group showed disorganized seminiferous tubules showing many spermatids with few spermatozoa (red arrows) (H&E × 100) and a Johnson score: Ref. [8] many spermatids with few spermatozoa. (**G**) Section of testis of CP+ Bilobetin 12-treated group showed average-sized seminiferous tubules showing many spermatozoa but disorganized spermatogenesis (blue arrows) (H&E × 100) and a Johnson score: Ref. [9] many spermatozoa but disorganized spermatogenesis.

The normal control rat testis section exhibited seminiferous tubules lined by layers of spermatogenic cells and filled with spermatozoa (Figure 6A). Figure 6B showed higher magnifications of normal testis and showed seminiferous tubule demonstrating complete layers of spermatogenesis consisting of layers of spermatogonia, spermatocytes, spermatids, and spermatozoa;a Johnson score was observed: Ref. [10] complete spermatogenesis with mature sperms. Figure 6C shows a section of testis of the Bilobetin 12-only treated group, which showed seminiferous tubules lined by layers of spermatogenic cells and filled with spermatozoa; a Johnson score was observed: Ref. [10] complete spermatogenesis with mature sperms. Figure 6D shows a section of testis of the Cisplatin-treated rat (positive control), which showed the destruction and disorganization of some seminiferous tubules. Figure 6E showed higher magnifications of CP-treated testis (positive control) showed seminiferous tubule showing many spermatocytes and no spermatids or spermatozoa; a Johnson score was observed: Ref. [5] showing many spermatocytes and no spermatids or spermatozoa. Figure 6F demonstrates the section of testis of the Cisplatin+ Bilobetin 6-treated group and showed disorganized seminiferous tubules showing many spermatids with few spermatozoa; a Johnson score was observed: Ref. [8] showing many spermatids with few spermatozoa. Figure 6G shows a section of testis of the Cisplatin+ Bilobetin 12-treated group and showed average-sized seminiferous tubules showing many spermatozoa but disorganized spermatogenesis; a Johnson score was observed: Ref. [9] showing many spermatozoa but disorganized spermatogenesis.

## 4. Discussion

One of the most serious problems in cancer treatment is testicular toxicity, which restricts the use and efficacy of antineoplastic drugs, such as CP. Mechanistically, CP attaches to the purine bases of DNA, causing DNA damage and indicating apoptotic or non-apoptotic cell death [26]. Cisplatin-induced serious testicular injury via suppressing testosterone production and inducing germ cell apoptosis. In addition, excessive oxidative stress has also been highlighted as the major cause of CP-induced testicular damage [6].

Bilobetin preventive efficacy against CP-induced testicular injury was investigated in this work. Bilobetin, on the other hand, significantly attenuated CP-induced testicular injury in rats, according to the findings. However, to the best of our knowledge, no previous studies on Bilobetin effects on CP-induced testicular toxicity have been previously reported.

Relative testes weight is a reliable normal spermatogenesis marker that is usually assessed in reproductive studies [27]. In the current investigation, cisplatin at a dose of 7 mg/kg drastically decreased relative testicular weight, which is consistent with earlier results [28,29]. In addition, Bilobetin treatment dramatically reduced testicular relative weight loss relative to the CP-treated group.

The major function of the testis is testosterone production [30]. Hence, testosterone measurements are regarded as a sensitive indicator for normal testicular function. Therefore, CP treatment induced a marked decrease in serum testosterone levels in this investigation, which agrees with prior studies [31,32].

The current study findings showed that Bilobetin might effectively prevent testosterone loss at a 12 mg/kg dose, suggesting its potential protective consequences on CP-induced testicular damage. This beneficial effect is confirmed by improving histopathological alterations induced by CP in testicular tissue and increasing Johnson scores by Bilobetin treatments.

In the present work, CP induced significant oxidative stress as indicated by a massive increase in testicular MDA levels and reduction in SOD activity in testicular tissue, and these results agree with previous reports [3,20,33,34,35]

Bilobetin treatment significantly attenuated oxidative stress by the notable reduction in lipid peroxidation levels and enhancement of SOD activity. These findings are consistent with other studies [17,36,37].

The transcription factor nuclear factor (erythroid-derived 2)-like 2 (Nrf2) safeguards against oxidative damage and inflammations [38] and is usually found in the cytoplasm, which is sequestered by Kelch-like ECH-associated protein 1 (Keap1). By serving as an adaptor molecule CUL-E3 ligase, Keap1 mediates Nrf2 ubiquitination and subsequent proteasomal degradation. Exposure to oxidative stress causes Keap1 to dissociate from the CUL-E3 ligase, which changes the cysteine residues of Keap1, resulting in Nrf-2 accumulation [39]. Consequently, Nrf2 is translocated into the nucleus where it increases antioxidant gene transcription [39,40].

Hence, Nrf2 activation is an excellent approach to mitigate oxidative stress. In this study, CP caused a significant reduction in Nrf-2 expression levels compared to the normal control and, thus, exacerbated oxidative stress conditions, and these results are in line with [5,41,42].

In addition, Bilobetin treatment markedly enhanced Nrf-2 gene expression levels and, hence, significantly ameliorated oxidative stress conditions. Moreover, in our work, CP triggered the noticeable regulation of Keap-1 expression levels that downregulates Nrf-2 expression in testicular tissue. Bilobetin administration markedly suppressed Keap-1 expression levels and, thus, may explain its enhancing effect on Nrf-2 expression. It is concluded that Bilobetin attenuates CP-induced oxidative stress, possibly by the modulation of the Nrf-2/Keap-1 signaling pathway. This is the first research study that shows that Bilobetin may provide protection against testicular injury caused by CP and investigated the possible underlying mechanisms of such protective effects.

In addition to the amelioration of oxidative stress, Nrf2 activation could substantially mitigate inflammation. It had a dramatic anti-inflammatory effect regulated by modulating NF-*κ*B, a master regulator of pro-inflammatory cytokines. It is reported that Nrf2 upregulation significantly diminished inflammatory responses in animal models [43,44,45,46].

Consequently, pharmacological enhancement of Nrf2 had the therapeutic potential for treating numerous diseases mediated by oxidative stress and inflammation [40].

In the current investigation, CP induced marked inflammation manifested by a significant downregulation of anti-inflammatory cytokine IL-10, and all findings agree with earlier research [34,35,47]. Bilobetin treatment also exhibited a significant anti-inflammatory effect. In addition, it significantly enhanced IL-10 testicular levels.

Nrf2 activation has also been reported to suppress NF*-κ*B-mediated transcription of adhesion molecules in endothelial cells, potentially via lowering free intracellular iron.

In the current study, CP induced a marked increase in VCAM expression levels, as evidenced previously [48]. This increase is significantly brought down by Bilobetin treatment, possibly via the enhancement of Nrf-2 expression levels.

The Keap1/Nrf2/ARE system functions as a central defensive mechanism against oxidative stress, which is implicated in the development of a variety of disorders. Bilobetin protects against CP-induced testicular injury in the current study via the mitigation of oxidative stress by disabling Keap-1and the upregulation of Nrf-2, which inhibits inflammatory response. Still, these results warrant further investigations to confirm these effects.

Chemotherapeutic drugs, for example, increase ROS in normal cells, causing inflammation, apoptosis, and oxidative stress [19,49]. Apoptosis is essential for normal homeostasis, but it can cause improper spermatogenesis or testicular injury if it occurs in the testis [50]. Intracellular stimuli such as oxidative stress stimulate the mitochondrial apoptotic pathway, which causes an imbalance in the Bcl-2 family’s expression, upregulating pro-apoptotic (Bax) and downregulating anti-apoptotic (Bcl-2), resulting in increased mitochondrial membrane permeability and a subsequent release of cytochrome-C into the cytosol [51]. The interaction of caspase-9 and apoptosome is stimulated by cytochrome C, which leads to caspase-3 activation, which is a critical factor in cell apoptosis [52].

In our investigation, testicular toxicity of CP is mediated through the activation of mitochondrial apoptosis, increasing Cytochrome-C in the cytosol and caspase-3 immunostaining, and this finding is consistent with [50,51]

Moreover, it is reported that apoptotic signaling is related to differences in apoptotic molecules involving p53 [53]. Whenever a cell is under apoptosis, p53 is activated, and cytochrome-C is released from the mitochondria, which activates caspase-3 [54,55].

The findings of this investigation revealed that CP induces apoptosis by activating certain apoptotic-regulated genes, including p53, Cytochrome-C and caspase-3, which were significantly upregulated compared to the normal control [19,50]. In contrast, P53, cytochrome-C, and caspase-3 were strongly decreased by Bilobetin treatments compared to CP-treated rats.

In the present investigation, Bilobetin exerted anti-apoptotic effects by reducing cytosolic Cytochrome-C, P53, and caspase-3 in CP-treated animal testes. Collectively, Bilobetin anti-apoptotic activity may be attributed to its antioxidative and anti-inflammatory effects.

Cell proliferation antigen Ki67 is employed chiefly in cancer prognosis and is a reliable marker for detecting a particular cell population [56]. In our investigation, CP administration inhibited the nuclear immunostaining of Ki67 in testes indicating, spermatogenic cells growth fraction decline, as reported earlier [21]. In addition, Bilobetin treatment increased Ki67 immunostaining, reflecting an increased proportion of proliferating cells. These data suggest the potential beneficial effects of Bilobetin administration in ameliorating CP-induced testicular toxicity via multiple effects.

The significant hallmarks of organ fibrosis are activating transforming growth factor-beta (TGFβ) and downstream alpha-smooth muscle actin (α-SMA). In addition, they often accumulate extracellular matrix accumulation that consequently leads to organ fibrosis [6].

Volkmann et al. [57] reported a significant relationship between increased lamina propria thickness and increased expression of α-SMA immunostaining with a marked disturbance in spermatogenesis score in testicular tissue.

This investigation found that cisplatin triggered a considerable upregulation of α-SMA expression relative to the control. On the contrary, Bilobetin administration caused a substantial reduction in α-SMA expression levels compared to CP-treated rats.

## 5. Conclusions

Bilobetin was isolated from *C. thouarsii* leaves for the first time. Bilobetin might be used as a potential approach for the attenuation of cisplatin-induced testicular damage because Bilobetin administration restored testosterone hormone production and improved the testes’ antioxidant properties via tge manipulation of Nrf-2/Keap-1 signaling. It also exhibited significant anti-inflammatory and anti-apoptotic effects by suppressing P53, Cytochrome-C release, and caspase-3 activation and restored the regenerative capacity of testes. The efficacy of Bilobetin in treating cisplatin-induced testicular damage should be demonstrated in future preclinical and clinical studies. The primary limitations of using bioflavonoids are their low oral bioavailability and poor solubility. In particular, if Bilobetin is formulated in some kind of drug delivery system, the results may be improved.

## Figures and Tables

**Table 1:** ^1^H-NMR and ^13^C-NMR (DMSO- *d_6_*, 500, and125 MHz) for Bilobetin.

	Compound I	
	δ-H	δ-C
2		163.5
3	6.93 (1H, s)	103.6
4		181.9
5		161.4
6	6.19 (1H, d, *J* = 2.5 Hz)	98.6
7		163.4
8	6.49 (1H, d, *J* = 2.5)	94.2
9		157.5
10		103.6
1′		122.6
2′	8.07 (1H, d, *J* =2.5)	128.3
3′		121. 6
4′		160.6
5′	7.48 (1H, d, *J* = 8.5)	111.7
6′	8.18 (1H, dd, *J* = 2.5, 8.5)	130.9
2″		164.3
3″	6.80 (1H, s)	102.5
4″		182.1
5″		160.6
6″	6.38 (1H, s)	98.9
7″		161.8
8″		103.7
9″		154.3
10″		103.7
1′′′		121.2
2′′′	7.51 (2H, d, *J* = 8.5),	128.0
3′′′	6.71 (2H, d, *J* = 8.5),	115.8
4′′′		161.1
5′′′	6.71 (2H, d, *J* = 8.5),	115.8
6′′′	7.51 (2H, d, *J* = 8.5)	128.0
4′-O-CH3	3.76	55.9

**Table 2:** Effects of Bilobetin treatment on testis/body weight ratio in Cisplatin-induced testicular toxicity in rats.

	Body Weight (gm)		Testis Weight (gm)	Testis/Body Weight Ratio
	Initial	Final		
Control	182.6 ± 1.95	198 ± 2.55	2.776 ± 126.0	1.397 ± 0.072
Bilobetin 12	180.5 ± 1.14	184.2 ± 9.03	2.57 ± 0.219	1.392 ± 0.1
Cisplatin	183.6 ± 1.82	167.4 ± 11.8 *	1.782 ± 0.168 *	1.038 ± 0.063 *
CP+ Bilobetin 6	182.4 ± 1.67	169.6 ± 12.1	2.1 ± 0.327	1.228 ± 0.125 ^#^
CP+ Bilobetin 12	183.5 ± 1.3	186.2 ± 6.38 ^#^	2.49 ± 0.105 ^#$^	1.338 ± 0.057 ^#^

Cisplatin induced testicular damage was induced by a single IP injection of cisplatin at 7 mg/kg at day 3. Rats were grouped randomly into control group; Bilobetin group was administered Bilobetin (12 mg/kg) IP daily for 10 days; untreated cisplatin group and cisplatin groups treated with Bilobetin (6,12 mg/kg, respectively) IP daily for 10 days and a single dose of IP injection of cisplatin 7 mg/kg at day 3. Data expressed as mean ± SD (n = 8/group). Significant difference vs. * respective control; ^#^ respective Cisplatin group; ^$^ respective CP+ Bilobetin 6 group each at *p* ˂ 0.05.

**Table 3:** Effects of Bilobetin treatment on testicular MDA level. Testicular SOD activity in Cisplatin-induced testicular toxicity in rats.

	Testicular MDA Content (nm/gm Tissue)	Testicular SOD Activity (U/mg Tissue)
Control	139.8 ± 2.86	2.81 ± 0.135
Bilobetin 12	140.8 ± 2.28	2.79 ± 0.09
Cisplatin	252 ± 5.33 *	1.38 ± 0.06 *
CP+ Bilobetin 6	183 ± 6.55 ^#^	1.724 ± 0.084 ^#^
CP+ Bilobetin 12	146.8 ± 3.03 ^#$^	2.58 ± 0.83 ^#$^

Cisplatin induced testicular damage was induced by a single IP injection of cisplatin at 7 mg/kg at day 3. Rats were grouped randomly into the control group. Bilobetin group was administered Bilobetin (12 mg/kg) IP daily for 10 days; untreated cisplatin group and cisplatin groups were treated with Bilobetin (6, 12 mg/kg, respectively) IP daily for 10 days and a single dose of IP injection of cisplatin 7 mg/kg at day 3. Data expressed as mean ± SD (*n* = 8/group). Significant difference vs. * respective control; ^#^ respective Cisplatin group; ^$^ respective CP+ Bilobetin 6 group each at *p* ˂ 0.05.

## Data Availability

The authors confirm that the data supporting this study are available within the article and/or its Supplementary Materials.

## Supplementary Materials

The following supporting information can be downloaded at: https://www.mdpi.com/article/10.3390/biomedicines10051134/s1, Table S1: Primers used and their sequence.
