# Supplementary material for: The Mechanistic Perspective of Bilobetin Protective Effects against Cisplatin-Induced Testicular Toxicity: Role of Nrf-2/Keap-1 Signaling, Inflammation, and Apoptosis
Source: Biomedicines. 2022 May 13;10(5):1134. doi: 10.3390/biomedicines10051134 (PMC9138600; doi:10.3390/biomedicines10051134)
Supplement: Supplementary file 1 [file biomedicines-10-01134-s001.zip › biomedicines-1717883-supplementary.pdf]

Supplementary Table S1. Primers used and their sequence

Gene	Probes
VCAM-1	5′-TCTTACCTGTGCGCTGTGAC-3′)<br>5′-ACATAAATGCCGGAATCGTC-3′)
Nrf2	ACACGGTCCACAGCTCATC<br>TGTCAATCAAATCCATGTCCTG
Keap1	ATTGGCTGTGTGGAGTTGC<br>CAGGTTGAAGAACTCCTCTTGC
P53	GTTCCGAGAGCTGAATGAGG<br>ACTTCAGGTGGCTGGAGTGA
IL10	5-CCA AGC CTT ATC GGA AAT GA-3<br>5-AGG G GA GAA ATC GAT GAC AG-3
α-SMA	TTG GAA AAG ATC TGG CAC CAC<br>GCA GTA GTC ACG AAG GAA TAG
GAPDH	5’- TGGAAGGACTCATGACCACA-3’<br>5’- ATGATGTTCTGGAGAGCCCC-3’
